# Facial and oral factors affecting smiles in young people

**DOI:** 10.1371/journal.pone.0360529

**Published:** 2026-10-09

**Authors:** Thuy Anh Vu Pham, Phong Phu Le, Vu Pham Phi Nguyen

**Affiliations:** Faculty of Odonto-Stomatology, University of Health Sciences, Vietnam National University, Ho Chi Minh City, Vietnam; Universidad Tecnica de Ambato, ECUADOR

## Abstract

**Objective:**

To characterize smile morphology and examine its associations with facial and oral parameters, with emphasis on factors associated with smile attractiveness in Vietnamese young adults.

**Materials and methods:**

Dynamic smiles of 490 Vietnamese individuals (mean age 21.04 ± 1.79 years) were recorded and analyzed. Categorical smile characteristics including anterior smile line, smile arc, upper lip curvature, most posterior tooth displayed, and upper teeth-lower lip relationship were assessed. Quantitative parameters comprised buccal corridor space, smile index, and dynamic smile symmetry. Multiple facial and dentofacial measurements were also obtained, including upper lip length, lip mobility, philtrum height, proportional indices, lip-line and occlusal plane cant, dental midline deviation, mouth size ratio, and incisal edge visibility. Participants were further classified according to smile attractiveness to identify associated determinants.

**Results:**

The anterior smile line, smile arc, and upper lip curvature demonstrated broad associations with several dentofacial parameters. The most posterior tooth displayed was significantly related to the philtrum height–upper lip length ratio and incisal edge visibility. Buccal corridor space and smile index showed comparatively limited correlations. Dynamic smile symmetry was significantly associated with upper lip length during smiling and mouth size ratio at rest. Several soft tissue and dentofacial variables were identified as contributing factors to smile attractiveness.

**Conclusion:**

Smile morphology in Vietnamese young adults is influenced by coordinated interactions between soft tissue dynamics and dentofacial proportions. These findings highlight population-specific characteristics and provide clinically relevant guidance for aesthetic evaluation and treatment planning in orthodontic and restorative practice.

## 1. Introduction

Smiling is a universal expression of happiness, yet their morphological characteristics vary across ethnic groups. Previous studies on smile dynamics and aesthetics have focused mainly on Caucasian populations, resulting in limited understanding of how ethnic differences shape smile expression and perceptions of beauty [1–3]. Consequently, applying smile characteristics identified in European-Americans to Asian populations, including Vietnamese individuals, may be inappropriate due to inherent ethnic variations. Smile aesthetics influence treatment outcomes and patient satisfaction and are shaped by demographic and individual preferences. Multiple smile characteristics, including the smile line, smile arc, buccal corridor space, upper lip curvature, smile index, and symmetry, have been associated with perceived attractiveness [4–6]. The smile line has been identified as an important parameter [7], and the smile arc is considered associated with smile aesthetics [8]. Additionally, buccal corridor width has been shown to affect smile attractiveness, with preferences varying across facial types and ethnic groups [9].

Besides the abovementioned smile characteristics, a number of oral factors may contribute to smile aesthetics, including the upper lip length, lip mobility, the philtrum height, the facial and dental midlines, the lip-line and occlusal plane cants, and the mouth size ratio. According to a previous study, people could detect differences between the anterior dental and facial midlines, particularly when these differences exceeded 2 mm [10]. Consistently, accurate midline alignment is important for smile aesthetics [11]. Moreover, oral factors such as the upper lip length, lip mobility, and the philtrum length can affect facial aesthetics and should be considered when analyzing facial beauty [12–14]. The lip-line and occlusal plane cants are the most noticeable characteristics in a patient with facial asymmetry [15]. It is crucial to understand these factors to develop more effective and satisfying treatment plans.

In recent years, researchers have begun addressing the ethnic gap by investigating smile characteristics in Chinese, Japanese, and Indian populations. These studies reported variations in the smile arc, upper lip curvature, and tooth display during smiling [4,16–20]. For instance, research on Chinese individuals showed a predominance of commissure and cuspid smiles [16], while similar differences in smile line and smile arc were observed in Indian populations [18,19]. However, most studies focused on young adults (18–25 years) and did not comprehensively examine broader age groups or the complex interactions between facial and oral structures influencing smile aesthetics.

In Vietnam, researchers have started to outline the specific characteristics of smile aesthetics. There are common features such as a high or average smile line, an upward curvature of the upper lip, and a parallel smile arc [5]. Moreover, smiles typically reveal teeth up to the second premolar. However, this study was limited by its relatively small sample size and did not provide a comprehensive evaluation of the morphological characteristics of smiles, particularly in relation to the dynamics between the mouth and face. Comprehensive evaluation of smile aesthetics requires assessment of key features such as the anterior smile line, smile arc, upper lip curvature, and buccal corridor space. Despite the recognized influence of ethnic variation on aesthetic perception, region-specific data remain limited, particularly for young Asian populations. To date, no study has provided a comprehensive analysis of smile morphology and related facial–oral factors in Vietnamese youth. Such data are essential for understanding population-specific characteristics and informing evidence-based aesthetic treatment planning.

In the present study, we examined various morphological aspects of smiles, including the anterior smile line, the smile arc, the upper lip curvature, the most posterior tooth displayed during smiling, the relationship between the upper teeth and the lower lip, the buccal corridor space, the smile index, and dynamic smile symmetry. We then investigated several facial and oral factors, namely the upper lip length, lip mobility, the philtrum height, the philtrum height to upper lip length ratio, the lip-line and occlusal plane cants, the correlation between the dental and facial midlines, the horizontal and vertical mouth size ratios, and the incisal edge visibility during smiling. Ultimately, this study aimed to provide information that may contribute to improved aesthetic assessment and treatment planning.

## 2. Materials and methods

### 2.1 Ethical approval

All participants were provided with detailed information about the study and gave written informed consent prior to enrollment. This research was approved by the Human Subjects Ethics Board of the University (No: 646/TĐHYKPNT-HDĐĐ on 21 March 2022), and was conducted in full accordance with the Helsinki Declaration of 1975, as revised in 2013.

### 2.2 Participant eligibility criteria

All participants underwent a standardized clinical examination to confirm eligibility according to the predefined inclusion criteria. Inclusion criteria were intact maxillary anterior dentition with mild or no crowding, Angle class I molar occlusion, no active caries or periodontal disease, no history of orthodontic or cosmetic treatment, and no maxillofacial trauma or lip abnormalities. Participants had no more than four missing teeth (excluding third molars) with intact maxillary anterior dentition to allow accurate evaluation of smile aesthetics. Professional scaling was performed one week prior to smile recording.

### 2.3 Study setting and recruitment period

Ethical approval was obtained in March 2022. From June 2022 to April 2023, eligible participants were progressively recruited from several constituent universities of Vietnam National University Ho Chi Minh City (VNU-HCM), Ho Chi Minh City, Vietnam. At the respective recruitment sites, eligible participants underwent clinical assessment and professional scaling, followed approximately one week later by standardized smile photography. Recruitment, scaling, and photography were conducted progressively throughout the recruitment period. After completion of recruitment and image acquisition in April 2023, the photographs were coded, standardized, and analyzed, followed by statistical analysis.

### 2.4 Sample size calculation

The minimum sample size was calculated using the formula for estimating a population proportion with specified precision. With parameters set at a 95% confidence level (Z = 1.96) and a 5% margin of error (d = 0.05) were used. A prevalence of 50% was assumed to provide the most conservative sample size estimate. The calculated minimum sample size was 385 participants. A total of 490 Vietnamese students (323 men and 167 women; mean age 21.04 ± 1.79 years; range, 18–29 years) who met the eligibility criteria were ultimately included in the study.

### 2.5 Photography acquisition and image standardization

The participants were instructed to sit up straight with their arms hanging naturally and their eyes looking straight at the camera. The head was adjusted so that the Frankfort plane was parallel to the floor throughout the imaging procedure. A ruler was placed with the smallest division of 1 mm close to the right side of the participant’s face so that the distance from the lens to the ruler and to the central incisor was equal.

A Canon EOS 6D digital camera (Canon Inc., Tokyo, Japan) with flash was mounted on a tripod 150 cm from the participant. The lens was positioned above the occlusal plane and oriented perpendicular to the sagittal plane against a plain white background. Participants were instructed to produce a natural, unforced smile. If the smile was considered unrepresentative, the photograph was repeated until a relaxed, reproducible smile was obtained. Full-face frontal photographs were obtained including the forehead, eyes, nose, lips, and chin, which was visible to allow assessment of facial reference lines and dentofacial measurements. Two images were captured for each participant: one with the lips at rest and one during a natural posed smile. All photographs were taken by a single trained operator.

After image acquisition, each photograph was assigned a unique identification code and imported into Adobe Photoshop CS6 (Adobe Systems, San Jose, CA, USA). The millimetre ruler positioned adjacent to the participants served as a calibration reference for converting pixel-based measurements into true distances (1 mm = known pixel distance). Brightness and contrast were adjusted uniformly, and the interpupillary line was aligned horizontally. Images were resized to a consistent resolution while maintaining the original aspect ratio. After calibration, each photograph was aligned according to the participant’s natural head position to ensure a standardized orientation. Images were then cropped while preserving all anatomical landmarks required for facial and smile analyses. The standardized images were subsequently used for the assessment of smile characteristics and facial and oral measurements prior to analysis.

### 2.6 Parameters investigated and examiner calibration

#### 2.6.1 Smile morphological characteristics (Fig 1).

Eight smile morphological characteristics were assessed: the anterior smile line, the smile arc, the upper lip curvature, the most posterior tooth displayed during smiling, the relationship between the upper teeth and the lower lip, the buccal corridor space, the smile index, and dynamic smile symmetry.

**Fig 1:**
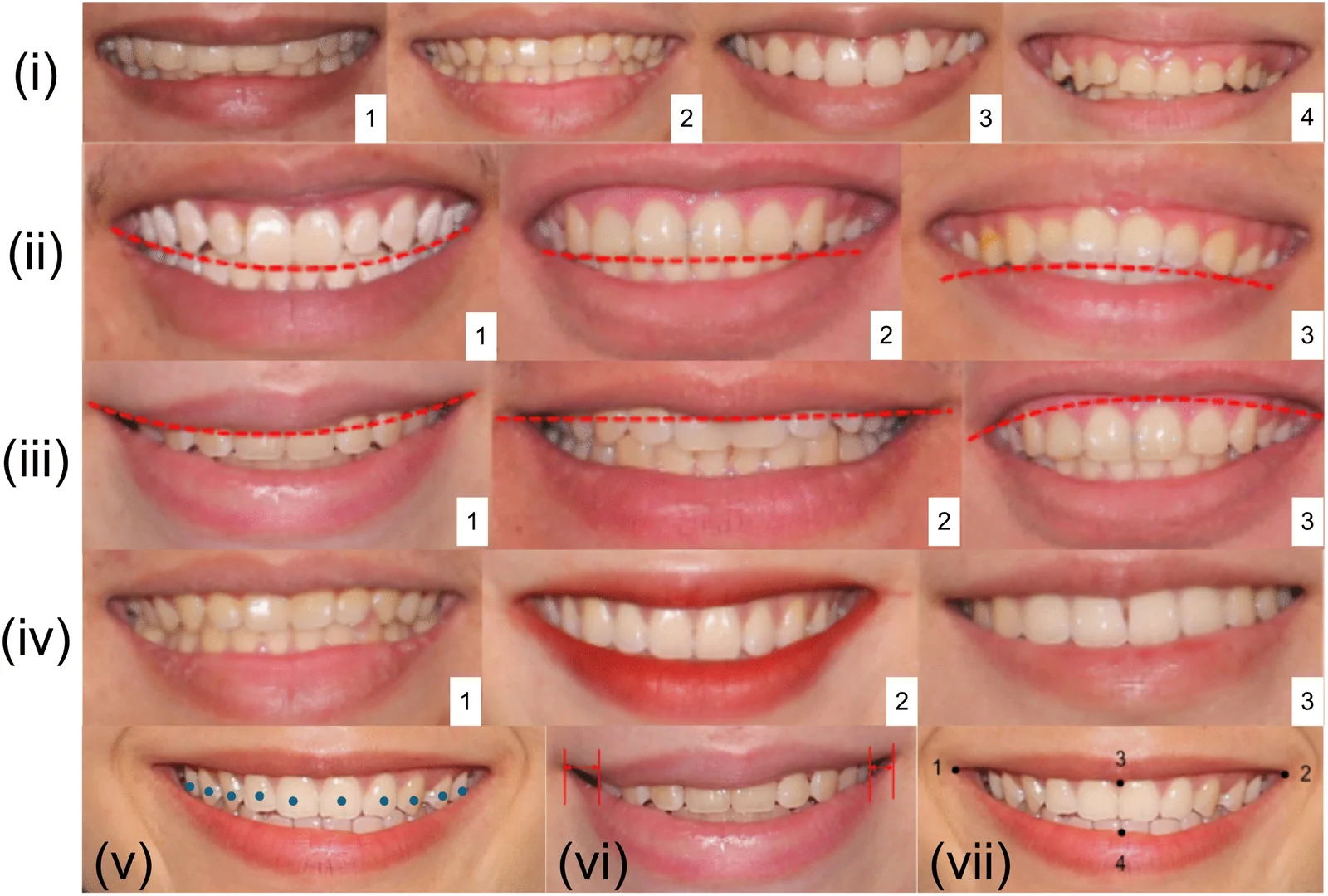
Representative examples of smile morphological features. (i) Anterior smile line: (1) low, (2) average, (3) high, and (4) very high. (ii) Smile arc: (1) parallel, (2) straight, and (3) reverse. (iii) Upper lip curvature: (1) upward, (2) straight, and (3) downward. (iv) Relationship between upper teeth and lower lip: (1) Not touch, (2) Touch, (3) Slightly covered. (v) Most posterior tooth displayed during smiling. (vi) Buccal corridor space. (vii) The reference points for calculating the smile index and dynamic smile symmetry.

- The anterior smile line is divided into four categories depending on the visible teeth and gingiva: (1) very high indicates > 2-mm marginal gingiva visible, (2) high indicates 0–2-mm marginal gingiva visible, (3) average indicates only the gingiva embrasure is visible, and (4): low indicates the gingiva is not visible [4].
- The smile arc is determined based on the relationship between the line drawn along the incisal edges of the maxillary central incisors to the cusp tips of the maxillary canines and the superior border of the lower lip. There are three categories: (1) a parallel smile arc means that the two lines are parallel to each other, (2) a straight smile arc means that the maxillary incisal edges and canine cusp tips are connected by a straight line with no curvature, and (3) a reverse smile arc means that the maxillary incisal edges and canine cusp tips form a reverse line relative to the superior border of the lower lip [4].
- The upper lip curvature describes the horizontal morphology of the inferior border of the upper lip based on the relationship between the corner of the mouth and the center of the inferior border of the upper lip. There are three categories: (1) an upward lip curvature indicates that the mouth corner is more than 1 mm above a horizontal line drawn through the center of the upper lip’s inferior border, (2) a straight lip curvature indicates that the mouth corner is at or within 1 mm above or below a horizontal line drawn through the center of the upper lip’s inferior border, and (3) a downward lip curvature indicates that the mouth corner is more than 1 mm below a horizontal line drawn through the center of the upper lip’s inferior border [4].
- The relationship between the upper teeth and the lower lip has three categories: not touch, touching, and slightly covering [2].
- The most posterior tooth displayed during smiling was classified as the canine, first premolar, second premolar, first molar, or second molar [4].
- Buccal corridor space was measured as the horizontal black space formed at the corners of the mouth during smiling [21].
- The smile index is the ratio of the horizontal distance between the outer commissure (i.e., the smile width) and the vertical distance between the inferior border of the upper lip and the superior border of the lower lip (i.e., the smile height) during smiling. It is calculated based on four reference points as [(1–2)/ (3–4)], where 1 is the right outer commissure, 2 is the left outer commissure, 3 is the center of the inferior border of the upper lip, and 4 is the center of the superior border of the lower lip [4].
- The dynamic smile symmetry refers to the uniformity of movement of the bilateral outer commissure in the horizontal and vertical direction. It is calculated based on the four reference points as [(1 − 3) + (1 − 4)]/ [(2 − 3) + (2 − 4)] [4].

#### 2.6.2 Smile attractiveness classification and subgroup division.

According to the results of previous research regarding the preferred smile features, participants’ smiles were separated into the attractive and unattractive subgroups based on their smile characteristics [4,5,22–25]. Participants were classified into the attractive smile subgroup if their smiles fulfilled all of the following predefined criteria: (1) a low, average, or high anterior smile line; (2) a parallel or straight smile arc; (3) an upward or straight upper lip curvature; (4) smile width extending to the second premolar or first molar; (5) the upper incisal edges either not touching or only slightly approaching the lower lip, without excessive coverage; and (6) a smile index greater than 5. The classification criteria for the two subgroups are presented in Table 1.

**Table 1:** Smile classification.

Smile features	Smile classification	
	Attractive	Unattractive
**Anterior smile line [4,5]**	Low, average, high	Very high
**Smile arc [5,23]**	Parallel, straight	Reverse
**Upper lip curvature [5,24]**	Upward, straight	Downward
**Most posterior tooth displayed during smiling [5,25]**	Second premolar, first molar	Canine, first premolar, second molar
**Relationship between the upper teeth and the lower lip [2,32]**	Touching, not touching	Slightly covering
**Smile index [4]**	> 5	< 5

#### 2.6.3 Facial and oral factors (Fig 2).

All recorded images were re-used to assess the facial and oral characteristics according to the following criteria:

**Fig 2:**
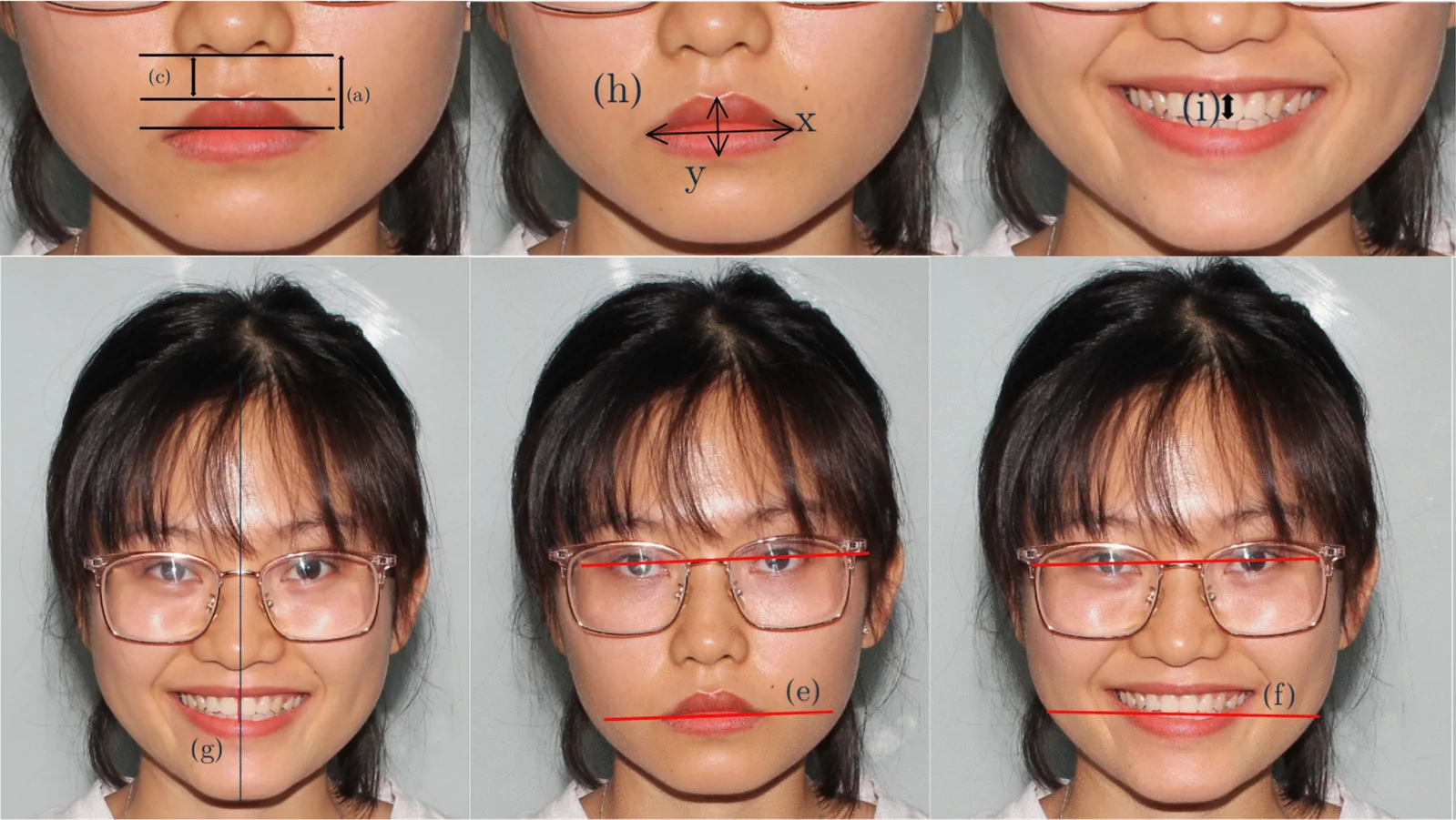
Representative examples of facial and oral factors. (a) Philtrum height, (c) Lip length, (e) Lip-line cant, (f) Occlusal plane cant, (g) Alignment between the dental and facial midlines, (h) Horizontal and vertical mouth size ratios, (i) Incisal edge of the incisors seen during smiling.

- Upper lip length – measured at rest and during smiling as the distance from the base of the nose to the lower edge of the upper lip [26,27];
- Lip mobility – calculated as the difference between upper lip length at rest and during smiling [26];
- Philtrum height – measured at rest and during smiling from the base of the nose to the upper edge of the upper lip, above the red lip border [27];
- Philtrum height to upper lip length ratio – determined at rest and during smiling by dividing philtrum height by upper lip length (unit %);
- Lip-line cant – calculated as the angle between the line passing through two corners of the mouth compared with the line passing through the eyes [28];
- Occlusal plane cant – during smiling, the occlusal plane is a straight line passing through the biting edge of two central incisors or the tip of the canine cusp. The occlusal plane cant of the anterior teeth is the angle between the occlusal plane and a straight line passing through the eyes [29];
- The dental and facial midlines – measured as the distance (if parallel) or angle (if not parallel) between the dental and facial midlines [30];
- The mouth size ratio – the width of the mouth horizontally is the distance between the two commissures of the mouth (x). The vertical height of the mouth is the distance between the upper edge of the upper lip and the lower edge of the lower lip (y). These proportions were measured at rest and during smiling (x/y);
- The incisal edge visibility during smiling – the distance from the lower edge of the upper lip to the incisal edge of the incisors [31].

#### 2.6.4 Examiner calibration.

Two examiners independently evaluated the smile characteristics according to the following criteria. Prior to the analysis, both examiners were calibrated using 30 representative photographs to standardize the assessment criteria. Inter-examiner reliability was assessed using Cohen’s kappa coefficient for the categorical variables (anterior smile line, smile arc, upper lip curvature, most posterior tooth displayed, relationship between upper teeth and lower lip) and the intraclass correlation coefficient for the quantitative measurements (buccal corridor space, smile index, dynamic smile symmetry, facial and oral factors). Inter-examiner reliability was good to excellent, with agreement coefficients ranging from 0.79 to 0.91.

### 2.7 Statistical analysis

#### 2.7.1 Analysis of smile morphological characteristics and facial and oral factors.

SPSS Statistics version 23.0 for Windows (IBM Corp., Armonk, NY, USA) was used to calculate the prevalence of the categorical smile morphological features (the anterior smile line, the smile arc, the upper lip curvature, the most posterior tooth displayed during smiling, the relationship between the upper teeth and the lower lip) and the mean ± standard deviation (SD) of the quantitative features (the buccal corridor space, the smile index, and dynamic smile symmetry, facial and oral factors) were determined. Gender differences were determined with the chi-squared test (for the categorical features) and the t-test (for the quantitative features).

#### 2.7.2 Correlation between smile morphological characteristics, facial and oral factors.

Pearson correlation coefficients were calculated to assess associations between smile morphological characteristics and facial and oral factors. All statistical tests were two-sided, and a p-value < 0.05 was considered statistically significant.

## 3. Results

### 3.1 Smile morphological characteristics and orofacial factors

For the anterior smile line, the majority of individuals fell into the average category (42.6%), followed by the low (32.7%), high (13.3%), and very high (11.4%) categories. The most prevalent smile arc was parallel (43.1%), followed by reverse (30.8%) and straight (26.1%). The majority of the participants had a downward curvature (55.9%), while 33.9% displayed a straight upper lip and 10.2% had an upward curvature. The second premolar was the most frequently displayed posterior tooth (58.6%), followed by the first molar (20.2%) and the first premolar (20.0%). The second molar and canine were displayed less, each accounting for less than 1% of the total sample. The majority of the participants (79.2%) exhibited no contact between their upper teeth and the lower lip. A smaller proportion demonstrated touching (17.1%), while a few participants had slightly covering (3.7%).

The mean ± SD values were 0.59 ± 0.47 for the buccal corridor space, 5.85 ± 1.48 for the smile index, and 1.01 ± 0.06 for dynamic smile symmetry. As shown in Table 2, significant sex differences were observed (p < 0.05) for the anterior smile line, the smile arc, the upper lip curvature, the most posterior tooth displayed during smiling, and dynamic smile symmetry. For the oral and facial factors, gender differences were observed in upper lip length, the philtrum height, the philtrum height to upper lip length ratio during smiling, the lip-line and occlusal plane cants, the distance between the dental and facial midlines, the mouth size ratio during smiling, and the incisal edge visibility during smiling.

**Table 2:** Differences in the smile morphological characteristics and orofacial factors according to gender (n = 490).

Smile characteristic and orofacial factors		Gender		Statistical analysis
		Male Number (%)	Female Number (%)	p*
**Anterior smile line**	Low	125 (38.7)	35 (21)	0.000
	Average	151 (46.7)	58 (34.7)	
	High	31 (9.6)	34 (20.4)	
	Very high	16 (5)	40 (24)	
**Smile arc**	Reverse	100 (31)	51 (30.5)	0.000
	Parallel	115 (35.6)	96 (57.5)	
	Straight	108 (33.4)	20 (12)	
**Upper lip curvature**	Downward	194 (60.1)	80 (47.9)	0.014
	Straight	95 (29.4)	71 (42.5)	
	Upward	34 (10.5)	16 (9.6)	
**Most posterior tooth displayed during smiling**	Canine	4 (1.2)	0 (0)	0.031
	First premolar	64 (19.8)	34 (20.4)	
	Second premolar	177 (54.8)	110 (56.9)	
	First molar	76 (23.5)	23 (13.8)	
	Second molar	2 (0.6)	0 (0)	
**Relationship between the upper teeth and the lower lip**	Touch	48 (14.9)	36 (21.6)	0.063
	Not touch	260 (80.5)	128 (76.6)	
	Slightly covering	15 (4.6)	3 (1.8)	
**Total**		323 (100)	167 (100)	
		**Mean ± standard deviation**	**Mean ± standard deviation**	p**
**Buccal corridor space (cm)**		0.6 ± 0.54	0.57 ± 0.29	0.454
**Smile index**		5.81 ± 1.57	5.93 ± 1.28	0.409
**Dynamic smile symmetry**		1.01 ± 0.07	0.99 ± 0.05	0.001
**Upper lip length at rest (cm)**		2.50 ± 0.20	2.28 ± 0.21	0.000
**Upper lip length during smiling (cm)**		1.93 ± 0.25	1.75 ± 0.23	0.000
**Lip mobility (cm)**		0.56 ± 0.19	0.53 ± 0.16	0.063
**Philtrum height at rest (cm)**		1.58 ± 0.23	1.44 ± 0.15	0.000
**Philtrum height during smiling (cm)**		1.16 ± 0.23	1.08 ± 0.20	0.001
**Philtrum height to upper lip length ratio at rest (%)**		63.20 ± 6.90	63.48 ± 4.30	0.583
**Philtrum height to upper lip length ratio during smiling (%)**		59.95 ± 10.13	62.04 ± 6.83	0.007
**Lip-line cant (**^**o**^)		1.45 ± 1.21	0.69 ± 0.74	0.000
**Occlusal plane cant (**^**o**^)		1.46 ± 1.40	1.71 ± 1.10	0.045
**Distance between the dental and facial midlines (cm)**		0.09 ± 0.09	0.11 ± 0.10	0.003
**Angle between the dental and facial midlines (**^**o**^)		1.48 ± 2.13	1.98 ± 2.942	0.052
**Mouth size ratio at rest**		2.44 ± 0.41	2.51 ± 0.36	0.068
**Mouth size ratio during smiling**		2.16 ± 0.31	2.28 ± 0.23	0.000
**The incisal edge visibility during smiling (cm)**		0.78 ± 0.27	0.87 ± 0.21	0.000

* Determined with the Pearson χ² test. ** Determined with a t-test.

### 3.2 Measurement of facial and oral factors on participants with attractive smiles

A total of 93 participants including 65 men and 28 women, fulfilled the predefined criteria for the attractive smile subgroup. Their facial and oral factors were then analyzed. Table 3 shows the mean ± SD values for each factor.

**Table 3:** The facial and oral factors of participants with attractive smiles (n = 93).

	Minimum	Maximum	Mean	Standard deviation
**Upper lip length at rest (cm)**	1.9	3.2	2.42	0.23
**Upper lip length during smiling (cm)**	1.2	2.5	1.87	0.26
**Lip mobility (cm)**	0.1	1	0.55	0.18
**Philtrum height at rest (cm)**	0.9	2.3	1.53	0.22
**Philtrum height during smiling (cm)**	0.5	1.8	1.13	0.22
**Philtrum height to upper lip length ratio at rest (%)**	45.00	79.31	63.29	6.13
**Philtrum height to upper lip length ratio during smiling (%)**	31.25	81.25	60.66	9.18
**Lip-line cant (**^**o**^)	0	4.7	1.19	1.13
**Occlusal plane cant (**^**o**^)	0	8.6	1.55	1.31
**Distance between the dental and facial midlines (cm)**	0	0.5	0.09	0.09
**Angle between the dental and facial midlines (**^**o**^)	0	9	1.65	2.45
**Mouth size ratio at rest**	1.48	4.55	2.46	0.40
**Mouth size ratio during smiling**	1.50	3.53	2.20	0.29
**The incisal edge visibility during smiling (cm)**	0	1.6	0.81	0.26

### 3.3 Comparison of demographic, facial, and oral characteristics between the attractive and unattractive smile groups

In Table 4, compared with the unattractive smile group, participants with attractive smiles exhibited significantly greater buccal corridor space, higher dynamic smile symmetry, greater philtrum height during smiling, a higher philtrum height-to-upper lip length ratio during smiling, smaller occlusal plane cant, a greater mouth size ratio during smiling, and greater maxillary incisal display during smiling (p < 0.05).

**Table 4:** Comparison of demographic, facial and oral characteristics between the attractive and unattractive smile groups (n = 490).

		Attractiveness		Statistical analysis
		Attractive	Unattractive	p*
**Demographic characteristics**		**Number (%)**	**Number (%)**	
**Gender**	Male	65 (69.89)	258 (64.99)	0.369
	Female	28 (30.11)	139 (35.01)	
**Total**		93 (100)	397 (100)	

**Facial and oral characteristics**		**Mean ± standard deviation**	**Mean ± standard deviation**	p**
**Buccal corridor space (cm)**		0.69 ± 0.48	0.57 ± 0.46	0.021
**Dynamic smile symmetry**		1.03 ± 0.07	1.00 ± 0.06	<0.001
**Upper lip length at rest (cm)**		2.42 ± 0.21	2.42 ± 0.23	0.947
**Upper lip length during smiling (cm)**		1.89 ± 0.21	1.86 ± 0.27	0.253
**Lip mobility (cm)**		0.53 ± 0.13	0.56 ± 0.19	0.064
**Philtrum height at rest (cm)**		1.53 ± 0.22	1.53 ± 0.22	0.970
**Philtrum height during smiling (cm)**		1.20 ± 0.24	1.12 ± 0.22	0.005
**Philtrum height to upper lip length ratio at rest (%)**		63.18 ± 5.17	63.32 ± 6.34	0.829
**Philtrum height to upper lip length ratio during smiling (%)**		62.87 ± 8.66	60.14 ± 9.23	0.010
**Lip-line cant (**^**o**^)		1.19 ± 0.83	1.19 ± 1.19	0.982
**Occlusal plane cant (**^**o**^)		1.14 ± 0.66	1.64 ± 1.40	<0.001
**Distance between the dental and facial midlines (cm)**		0.08 ± 0.10	0.10 ± 0.09	0.071
**Angle between the dental and facial midlines (**^**o**^)		1.30 ± 2.09	1.73 ± 2.52	0.125
**Mouth size ratio at rest**		2.42 ± 0.44	2.47 ± 0.39	0.257
**Mouth size ratio during smiling**		2.32 ± 0.27	2.18 ± 0.29	<0.001
**The incisal edge visibility during smiling (cm)**		0.69 ± 0.18	0.84 ± 0.27	<0.001

* Determined with the Pearson χ² test. ** Determined with a t-test.

### 3.4 Differences in facial and oral factors according to gender within attractive and unattractive smile subgroups

Additional analyses were performed to compare male and female participants separately within the attractive and unattractive smile subgroups (Table 5). Among participants with attractive smiles, significant sex differences were observed in several smile characteristics and facial and oral factors, including the anterior smile line, smile arc, upper lip curvature, relationship between the upper teeth and lower lip, smile index, upper lip length, philtrum height during smiling, philtrum-to-upper-lip-length ratios, occlusal plane cant, distance between the dental and facial midlines, and incisal edge visibility during smiling (p < 0.05).

**Table 5:** Differences in facial and oral factors according to gender within attractive and unattractive smile subgroups.

Smile characteristic and orofacial factors		Attractiveness					
		Attractive (n = 93)			Unattractive (n = 397)		
		Male Number (%)	Female Number (%)	p*	Male Number (%)	Female Number (%)	p*
**Anterior smile line**	Low	45 (69.23)	2 (7.14)	< 0.001	80 (31.01)	33 (23.74)	< 0.001
	Average	20 (30.77)	24 (85.71)		131 (50.78)	34 (24.46)	
	High	0 (0)	2 (7.14)		31 (12.02)	32 (23.02)	
	Very high	0 (0)	0 (0)		16 (6.20)	40 (28.78)	
**Smile arc**	Reverse	0 (0)	0 (0)	< 0.001	100 (38.76)	51 (36.69)	< 0.001
	Parallel	32 (49.23)	26 (92.86)		83 (32.17)	70 (50.36)	
	Straight	33 (50.77)	2 (7.14)		75 (29.07)	18 (12.95)	
**Upper lip curvature**	Downward	0 (0)	0 (0)	0.276	194 (75.19)	80 (57.55)	< 0.001
	Straight	52 (80)	25 (89.29)		43 (16.67)	46 (33.09)	
	Upward	13 (20)	3 (10.71)		21 (8.14)	13 (9.35)	
**Most posterior tooth displayed during smiling**	Canine	0 (0)	0 (0)	0.072	4 (1.55)	0 (0)	0.085
	First premolar	0 (0)	0 (0)		64 (24.81)	34 (24.46)	
	Second premolar	47 (72.31)	25 (89.29)		130 (50.39)	85 (61.15)	
	First molar	18 (27.69)	3 (10.71)		58 (22.48)	20 (14.39)	
	Second molar	0 (0)	0 (0)		2 (0.78)	0 (0)	
**Relationship between the upper teeth and the lower lip**	Touch	0 (0)	7 (25)	< 0.001	48 (18.60)	15 (10.79)	0.022
	Not touch	65 (100)	21 (75)		195 (75.58)	121 (87.05)	
	Slightly covering	0 (0)	0 (0)		15 (5.81)	3 (2.16)	
**Total**		65 (100)	28 (100)		258 (100)	139 (100)	
		**Mean ± standard deviation**	**Mean ± standard deviation**	p**	**Mean ± standard deviation**	**Mean ± standard deviation**	p**
**Buccal corridor space (cm)**		0.74 ± 0.53	0.57 ± 0.31	0.061	0.56 ± 0.53	0.57 ± 0.29	0.858
**Smile index**		6.26 ± 0.74	6.95 ± 0.81	< 0.001	5.70 ± 1.71	5.73 ± 1.27	0.868
**Dynamic smile symmetry**		1.04 ± 0.07	1.01 ± 0.06	0.129	1.01 ± 0.06	0.99 ± 0.05	0.010
**Upper lip length at rest (cm)**		2.47 ± 0.21	2.32 ± 0.19	0.002	2.50 ± 0.20	2.27 ± 0.21	< 0.001
**Upper lip length during smiling (cm)**		1.93 ± 0.20	1.82 ± 0.20	0.020	1.94 ± 0.25	1.73 ± 0.24	< 0.001
**Lip mobility (cm)**		0.54 ± 0.12	0.50 ± 0.14	0.193	0.57 ± 0.20	0.54 ± 0.16	0.110
**Philtrum height at rest (cm)**		1.55 ± 0.23	1.50 ± 0.19	0.357	1.59 ± 0.23	1.43 ± 0.14	< 0.001
**Philtrum height during smiling (cm)**		1.25 ± 0.24	1.08 ± 0.19	< 0.001	1.14 ± 0.23	1.09 ± 0.20	0.032
**Philtrum height to upper lip length ratio at rest (%)**		62.49 ± 5.09	64.79 ± 5.08	0.049	63.38 ± 7.28	63.21 ± 4.10	0.775
**Philtrum height to upper lip length ratio during smiling (%)**		64.52 ± 8.72	59.04 ± 7.31	0.005	58.79 ± 10.14	62.64 ± 6.59	< 0.001
**Lip-line cant (**^**o**^)		1.30 ± 0.86	0.95 ± 0.72	0.062	1.49 ± 1.28	0.64 ± 0.74	< 0.001
**Occlusal plane cant (**^**o**^)		1.03 ± 0.62	1.38 ± 0.70	0.020	1.57 ± 1.52	1.78 ± 1.15	0.156
**Distance between the dental and facial midlines (cm)**		0.10 ± 0.10	0.04 ± 0.07	0.007	0.08 ± 0.08	0.13 ± 0.10	< 0.001
**Angle between the dental and facial midlines (**^**o**^)		1.26 ± 2.29	1.40 ± 1.56	0.756	1.54 ± 2.09	2.10 ± 3.14	0.060
**Mouth size ratio at rest**		2.41 ± 0.45	2.43 ± 0.42	0.838	2.44 ± 0.40	2.52 ± 0.35	0.056
**Mouth size ratio during smiling**		2.31 ± 0.30	2.34 ± 0.21	0.559	2.13 ± 0.30	2.27 ± 0.24	< 0.001
**The incisal edge visibility during smiling (cm)**		0.61 ± 0.13	0.88 ± 0.12	< 0.001	0.82 ± 0.28	0.87 ± 0.22	0.064

* Determined with the Pearson χ² test. ** Determined with a t-test.

Among participants with unattractive smiles, significant sex differences were also observed across several smile characteristics and facial and oral factors, including the anterior smile line, smile arc, upper lip curvature, relationship between the upper teeth and lower lip, dynamic smile symmetry, upper lip length, philtrum height, philtrum-to-upper-lip-length ratio during smiling, lip-line cant, distance between the dental and facial midlines, and mouth size ratio during smiling (p < 0.05).

### 3.5 Correlation between the oral factors and smile morphological characteristics

Table 6 shows the correlations between the facial and oral factors and smile morphological characteristics. The anterior smile line was significantly associated with all of the oral factors except the philtrum height to the upper lip length ratio during smiling. The smile arc correlated with most of the oral factors except the philtrum height during smiling, the philtrum height to the upper lip length ratio, and the angle between the dental and facial midlines. The upper lip curvature also correlated with almost all of the oral factors, except the upper lip length and the philtrum height at rest, the lip-line cant, and the distance between the dental and facial midlines.

**Table 6:** Correlations between the facial and oral factors and the smile morphological characteristics (n = 490).

Oral factors Morphological characteristics of smiles	Upper lip length at rest	Upper lip length during smiling	Lip mobility	Philtrum height at rest	Philtrum height during smiling	Philtrum height to upper lip length ratio at rest	Philtrum height to upper lip length ratio during smiling	Lip-line cant	Occlusal plane cant	Distance between the dental and facial midlines	Angle between the dental and facial midlines	Mouth size ratio at rest	Mouth size ratio during smiling	The incisal edge visibility during smiling
**Anterior smile line** ^**a**^	**0**	**0**	**0**	**0.017**	**0**	0.125	**0.026**	**0.027**	**0.001**	**0**	**0**	**0.002**	**0.033**	**0**
**Smile arc** ^**a**^	**0**	**0.001**	**0.027**	**0.001**	0.161	0.308	0.126	**0**	**0**	**0.004**	0.387	**0.002**	**0**	**0.002**
**Upper lip curvature** ^**a**^	0.876	**0**	**0**	0.281	**0**	**0.016**	**0.001**	0.406	**0.002**	0.587	**0.003**	**0**	**0**	**0**
**Most posterior tooth displayed during smiling** ^**a**^	0.18	0.551	0.052	0.432	0.278	**0.002**	**0.033**	**0**	**0**	0.07	**0**	0.939	0.819	**0**
**Relationship between the upper teeth and the lower lip** ^**a**^	0.276	0.156	**0**	**0**	**0.008**	**0**	**0**	**0**	**0**	**0.001**	**0.001**	**0**	**0**	**0.002**
**Buccal corridor space** ^**b**^	.070	−.043	**.149** ^**^	**−.101** ^*^	−.002	**−.222** ^**^	.015	−.018	−.050	−.055	.020	.065	**.217** ^**^	.067
**Smile index** ^**b**^	−.043	**.317** ^**^	**−.504** ^**^	.064	**.227** ^**^	**.136** ^**^	.012	**−.122** ^**^	**.183** ^**^	−.018	**−.093** ^*^	**.140** ^**^	**.592** ^**^	**−.484** ^**^
**Dynamic smile symmetry** ^**b**^	.063	**.122** ^**^	**−.094** ^*^	**−.140** ^**^	.029	**−.269** ^**^	−.086	−.081	**.111** ^*^	.067	.003	**−.303** ^**^	.028	−.077

^a^: Assessed with analysis of variance; ^b^: Assessed with Pearson correlation analysis; ** The correlation is significant (p < 0.01);

* The correlation is significant (p < 0.05).

There were correlations between the most posterior tooth displayed during smiling and the philtrum height to the upper lip length ratio, the lip-line and occlusal plane cants, the angle between the dental and facial midlines, and the incisal edge visibility during smiling. The relationship between the upper teeth and the lower lip correlated with all of the oral factors except the upper lip length. On the other hand, the buccal corridor space correlated with just four oral factors, namely lip mobility, the philtrum height at rest, the philtrum height to the upper lip length ratio at rest, and the mouth size during smiling. The smile index correlated with most of the oral factors except the upper lip length and the philtrum height at rest, the philtrum height to upper lip length ratio during smiling, and the distance between the dental and facial midlines. Finally, dynamic smile symmetry correlated with six oral factors: the upper lip length during smiling, lip mobility, the philtrum height at rest, the philtrum height to upper lip length ratio at rest, the occlusal plane cant, and the mouth size ratio at rest.

## 4. Discussion

This study identifies three key findings among Vietnamese youth: (1) a predominance of average anterior smile lines and parallel smile arcs, (2) a notably high prevalence of downward upper lip curvature within this population, and (3) broad correlations between smile morphology and multiple dentofacial parameters, particularly occlusal plane cant, philtrum height during smiling, maxillary incisal display, and mouth size ratio during smiling. These parameters were associated with a wide range of smile characteristics, suggesting that they may contribute in smile appearance. Because some of these variables may be modified through orthodontic treatment, restorative procedures, prosthodontic rehabilitation, or surgical interventions, they may serve as useful clinical indicators during smile analysis and treatment planning. The findings also emphasize that smile aesthetics should be evaluated comprehensively, considering the interaction of multiple soft-tissue and dentofacial components rather than relying on a single morphological feature. This study provides a comprehensive analysis of smile morphology in Vietnamese individuals, revealing significant variations in the anterior smile line, smile arc, and upper lip curvature. An average anterior smile line and a parallel smile arc were more prevalent than reported in a German population [2]. Previous studies have shown that a parallel (“consonant”) smile arc is generally perceived as more youthful and attractive [22], and this feature may therefore be considered during orthodontic treatment planning. In contrast to the more common upward lip curvature reported in Western populations [3], over half of our participants exhibited a downward upper lip curvature. These findings support the need for population-specific reference values when evaluating smile aesthetics in Vietnamese individuals.

Compared with studies conducted in Germany [2] and Spain [3], the prevalence of a high smile line in our Vietnamese sample suggests distinct dentofacial characteristics. We observed a higher proportion of average smile lines and fewer high smile lines than previously reported. These findings are clinically relevant in evaluating excessive gingival display. The distribution of the most posterior tooth displayed during smiling was comparable to previous reports [2,5], with the second premolar most frequently visible, although the canine and second molar were occasionally observed. Most participants showed no contact between the upper teeth and lower lip, consistent with earlier studies [2,32]. The mean buccal corridor space was 0.59; direct comparison was limited due to differences in measurement methods, as prior studies primarily reported ratios rather than absolute values.

To further validate the smile classification criteria, the facial and oral characteristics of the attractive and unattractive smile groups were compared. The two groups did not differ significantly in sex distribution, indicates that the proportions of men and women classified as attractive or unattractive did not differ significantly in this sample. However, several facial and oral measurements differed significantly between the groups. The higher dynamic smile symmetry observed in the attractive group supports the concept that balanced bilateral lip movement during smiling enhances smile harmony and facial aesthetics. Significant differences were also observed in several soft tissue measurements. Together, these findings indicate that smile attractiveness is associated not only with the predefined smile characteristics but also with soft tissue behavior and dental relationships. These results support the use of a comprehensive, criteria-based approach for smile assessment rather than relying on individual esthetic parameters.

The sex-stratified analysis provided additional evidence that sex-related differences in morphological features and orofacial factors were observed within both smile-attractiveness subgroups. Within the attractive smile subgroup, males predominantly exhibited a low anterior smile line (69.23%) and a higher frequency of straight smile arcs (50.77%), whereas females more frequently displayed an average anterior smile line (85.71%) and parallel smile arcs (92.86%). Females also showed a greater proportion of upper teeth–lower lip contact (25% versus 0% in males) and significantly higher smile index values (6.95 ± 0.81 versus 6.26 ± 0.74, p < 0.001). Soft-tissue measurements further differed by gender: males had longer upper lip lengths both at rest and during smiling, greater philtrum height during smiling, and larger midline deviations, while females demonstrated greater incisal edge visibility (0.88 ± 0.12 cm versus 0.61 ± 0.13 cm, p < 0.001). These patterns suggest that, even among individuals meeting the predefined criteria for an attractive smile, males and females achieve aesthetic outcomes through distinct morphological combinations. In particular, the preference for parallel smile arcs and higher smile indices among attractive female smiles aligns with previously reported aesthetic ideals, whereas attractive male smiles appear to tolerate straighter arcs and lower gingival display. In the unattractive subgroup, gender differences were more numerous and pronounced. Significant disparities were found for anterior smile line, smile arc, upper lip curvature, relationship between the upper teeth and lower lip, dynamic smile symmetry, upper lip length, philtrum height, several proportional indices, lip-line cant, midline distance, and mouth size ratio during smiling. Notably, unattractive males showed a markedly higher prevalence of downward upper lip curvature (75.19%) compared with unattractive females (57.55%), and females exhibited a higher frequency of very high smile lines. These findings indicate that the morphological features contributing to reduced smile attractiveness also differ systematically by gender. Therefore, aesthetic evaluation and treatment planning should take gender-specific characteristics into account rather than applying uniform standards.

The mean smile index (5.85) in our cohort was within the attractive range reported in previous studies [33,34], and comparable to values observed in Chinese and Japanese populations [4,20]. Dynamic smile symmetry was also consistent with accepted aesthetic thresholds [4]. In our sample, women tended to display more traits associated with the attractive subgroup, particularly regarding smile arc, upper lip curvature, and smile index. Regarding dentofacial parameters, the mean upper lip length, lip mobility, and philtrum height were comparable to previously reported values [12,27]. Lip-line and occlusal plane cant measurements were also within ranges reported in previous studies [28,35]. Midline discrepancies and incisal display were also within clinically acceptable limits [31,36]. Direct comparisons for some ratios were limited due to scarce reference data. Few studies have examined comprehensive correlations between smile characteristics and dentofacial factors [37]. Compared with previous investigations, our study incorporated a larger set of variables and frontal-view analysis, providing a broader evaluation of smile morphology in Vietnamese individuals.

This study has several limitations. First, the participants consisted exclusively of Vietnamese young adults; therefore, the findings may not be generalizable to children, older adults, or other ethnic populations. Second, because this was a cross-sectional study, the observed associations between smile morphology and facial and oral factors should not be interpreted as causal relationships. Another limitation was the unequal distribution of male and female participants in the study sample. Although sex-specific analyses were performed, the smaller number of female participants may limit the broader generalizability of the female-specific findings, particularly regarding smile aesthetics. Future studies involving participants from different regions, broader age groups, and multiple ethnic populations, would help validate and extend the present findings.

## 5. Conclusion

This study provides a comprehensive dataset on smile morphology, facial and oral factors among Vietnamese young adults, with a particular emphasis on attractive smiles. This contribute to the understanding of smile characteristics in young Vietnamese adults and may inform future research and individualized aesthetic treatment planning.

## Supporting information

S1 Dataset Anonymized dataset containing the variables used for all statistical analyses reported in this study. (XLSX)
